# Cap polyposis treated with laparoscopic-assisted total proctocolectomy and ileal J-pouch anal anastomosis: a case report

**DOI:** 10.1186/s40792-021-01214-7

**Published:** 2021-06-29

**Authors:** Tomohiro Minagawa, Hiroki Ikeuchi, Kurando Kusunoki, Ryuichi Kuwahara, Yuki Horio, Takako Kihara, Seiichi Hirota, Motoi Uchino

**Affiliations:** 1 Department of Inflammatory Bowel Disease Surgery, Hyogo College of Medicine, 1-1 Mukogawa-cho, Nishinomiya, 663-8501 Japan; 2 Department of Surgical Pathology, Hyogo College of Medicine, 1-1 Mukogawa-cho, Nishinomiya, 663-8501 Japan

**Keywords:** Cap polyposis, Ileal pouch anal anastomosis, Laparoscopic surgery, Background

## Abstract

**Background:**

Cap polyposis (CP) is extremely rare in Japan, and there is no established cure. We report a case in which CP was improved by surgical treatment.

**Case presentation:**

A 48-year-old man was investigated at a local hospital because of diarrhea and bloody stools in 2018. The patient was treated with metronidazole for suspected amoebic dysentery, but his symptoms did not improve. Subsequent close examination revealed possible CP, but treatment with 5-aminosalicylic acid and a steroid enema had no effect. The patient was then referred to our hospital. The bloody stools, diarrhea, and abdominal pain worsened despite medical treatment, so laparoscopic-assisted total proctocolectomy and ileal J-pouch anal anastomosis with ileostomy were performed. CP has no known cause or established treatment, but *Helicobacter pylori* (HP) infection has been reported in many CP cases in Japan, and HP eradication is often successful. This patient was HP-negative and did not improve with antimicrobial treatment, but the symptoms improved after surgery.

**Conclusions:**

Even after surgery, CP recurrence reportedly occurs within a short period in many cases. However, our patient has had no signs of CP recurrence during 1 year of follow-up.

Cap polyposis (CP) is an inflammatory disease of the large intestine that was first reported in 1985 and has characteristic clinical, endoscopic, and histopathological findings [1]. Some cases of CP have been reported in Japan, but it is an extremely rare disease that is difficult to differentiate from other inflammatory bowel diseases. The etiology of CP is unclear and there is no established cure. Even after surgery, recurrence within a short period is common [2, 3]. In Japan, it has been reported that *Helicobacter pylori* (HP) eradication is successful in the treatment of CP [2, 4]. Herein, we report a case in which surgical treatment was effective for CP resistant to medical treatment.

## Case presentation

A 48-year-old man presented at a local hospital because of diarrhea and bloody stools in 2018 and was started on probiotics. There was no relevant medical history and/or family history. The patient’s symptoms did not improve, and he represented at the local hospital 2 months later. Total colonoscopy (TCS) revealed wart-like erosions resembling octopus suckers from the sigmoid colon to the rectum, suggesting amoebic dysentery. However, stool cultures and biopsied colonic mucosa did not show dysenteric amoebae at that time. The patient was started on oral metronidazole (MNZ), but the symptoms did not improve and there were no improvements seen on repeat TCS performed 2 months later. The patient was also negative for amoebic dysentery antibody at that time and CP was suspected based on the TCS findings, but a definitive diagnosis was not reached. Administration of oral 5-aminosalicylic acid (5-ASA) and a steroid enema also resulted in no improvements in the symptoms. The patient was then referred to the Department of Internal Medicine of our hospital for further examination and medical treatment.

**Fig. 1:**
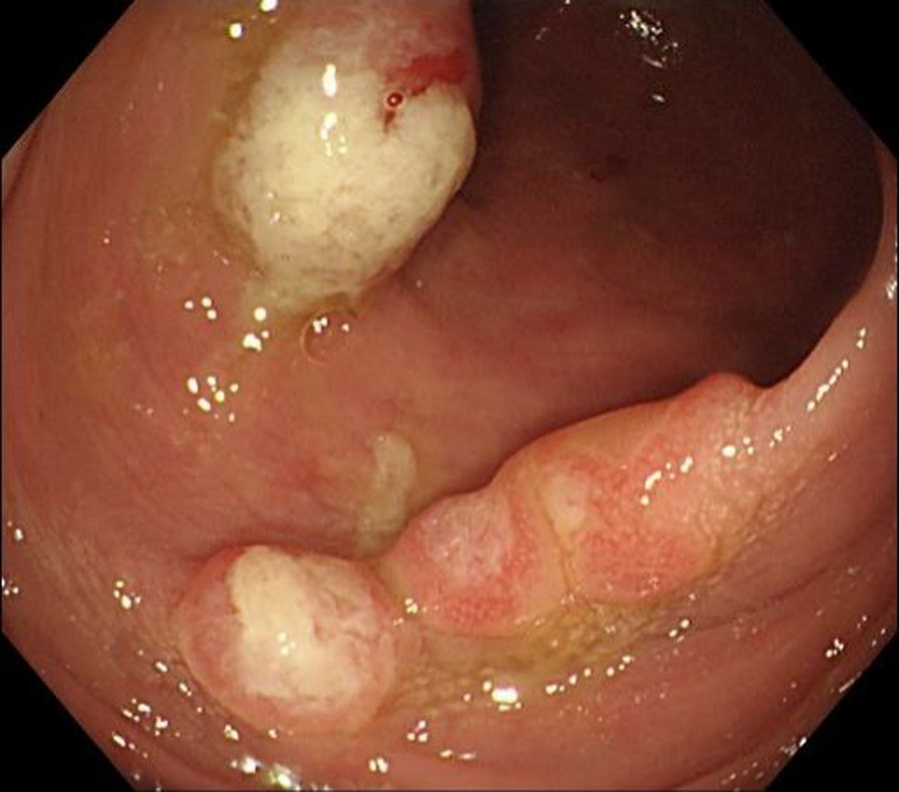
Total colonoscopy examination. The mucosa is reddened and there are multiple raised lesions from the middle transverse colon to the rectum. There is white moss on the top of the raised lesions

TCS performed at our hospital revealed reddened mucosa and multiple raised lesions from the middle of the transverse colon to the rectum; these lesions were covered with white moss (Fig. 1). Pathological examination showed inflammatory cell infiltration in the interstitium, and granulation tissue in the superficial layer, consistent with CP. A diagnosis of CP was made based on the clinical symptoms, and endoscopic and pathological findings. The patient was negative for HP. However, as all other treatments had been ineffective and HP eradication has been reported to be effective in treating CP in Japan, a proton pump inhibitor, amoxicillin, and clarithromycin were administered. The symptoms persisted despite these treatments, and the patient’s weight reduced from 78 to 60 kg.

In May 2019, the patient was admitted to the Department of Internal Medicine of our hospital due to exacerbation of his symptoms. Laboratory tests showed hypoalbuminemia, mild elevations in the C-reactive protein level and erythrocyte sedimentation rate, and a normal white blood cell count. There were no abnormal stool culture findings on admission. The patient was resistant to medical treatment and was admitted to our department, because he wished to undergo surgery.

**Fig. 2:**
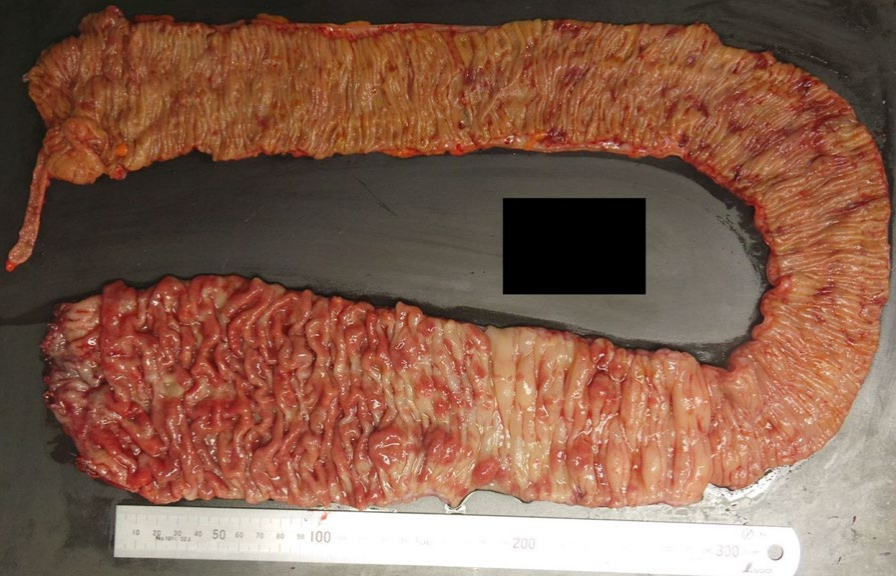
Examination of specimens after total colectomy. The mucosa is reddened and there are multiple raised lesions from the descending colon to the rectum

**Fig. 3:**
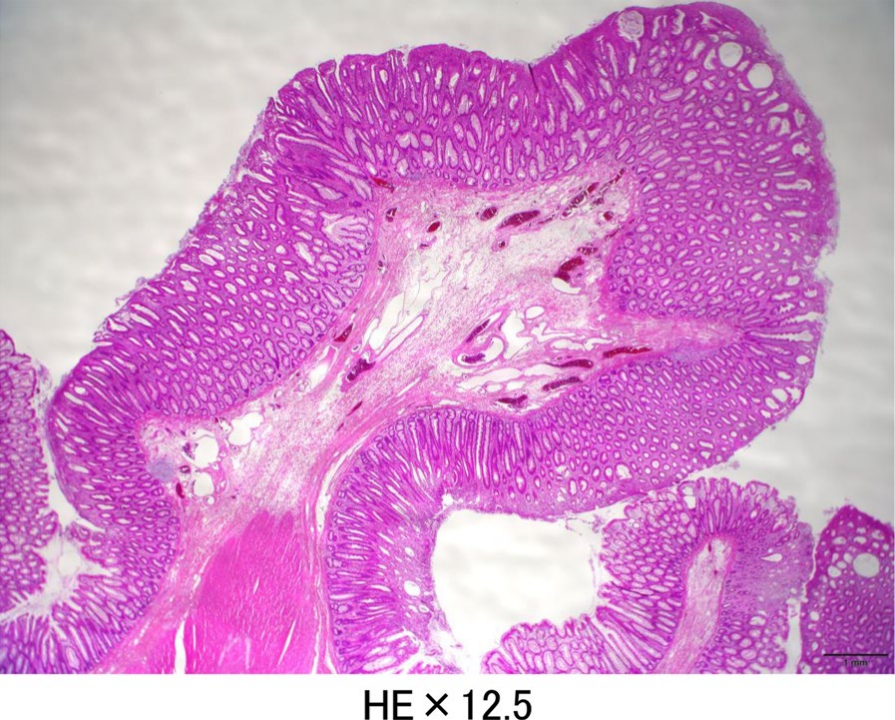
Histopathological examination. There are multiple raised lesions from the descending colon to the rectum. Each lesion is covered with granulation tissue with inflammatory exudate in a hat-like manner, with hyperplasia of the crypt epithelium

Intraoperatively, we found polyps from the rectum to the middle of the transverse colon, and inflammation in the anal canal. Therefore, we performed a laparoscopic-assisted total proctocolectomy and J-type ileal pouch anal anastomosis (IPAA) with ileostomy (Fig. 2). After the operation, his symptoms rapidly improved, and he started eating on postoperative day 3. He was discharged without problems on postoperative day 14. Postoperative pathological examination of the resected specimen showed multiple elevated lesions from the descending colon to the rectum. The apex of each lesion was capped with granulation tissue with inflammatory exudate, and the crypt epithelium was hyperplastic, consistent with CP (Fig. 3). There was no recurrence of symptoms or polyps in the ileal pouch, and stoma closure was performed 3 months after surgery. Approximately 1 year has passed since the stoma closure, and there has been no recurrence observed.

## Discussion

There are no definitive diagnostic criteria for CP. CP is a condition in which nonspecific inflammatory polyps develop mainly in the rectum and sigmoid colon, and the symptoms of CP are mucus secretion, diarrhea, bloody stools, and sometimes abdominal pain [2]. Blood tests often show hypoproteinemia, and the characteristic endoscopic findings of CP include multiple broad-based bulging lesions from the rectum to the sigmoid colon, with mucus attachment on the surface [2]. Pathological examination shows that the mucosal surface of the lesions is covered with inflammatory granulation tissue, which is called Cap [2]. The diagnosis of CP in Japan is based on the abovementioned characteristic symptoms and examination findings, which were used to diagnose CP in the present patient.

It has been theorized that the mechanism of CP may be mechanical stimulus or infection, but the definitive cause remains unknown [1, 2, 4]. There is currently no established treatment for CP, although various treatments have been used. 5-ASA is often ineffective in treating CP, while steroids cause temporary improvements followed by relapse [2, 5]. Although there are reports that MNZ is effective for CP [6], there are also reports that MNZ is ineffective [1, 2]; the response rate of MNZ is 28.6% [5]. Similarly, some reports that infliximab is effective in treating CP [7], while others report that it is ineffective [8]. In Japan, HP eradication has reportedly been successful in treating many cases of CP [2, 4]. In our case, various treatments such as MNZ, 5-ASA, steroids, and HP eradication therapy were not effective. Most of the previously reported cases of successful treatment of CP via HP eradication have involved patients with confirmed HP infection, and the relationship between HP infection and CP is drawing attention [2, 4]. However, there are reports of CP in patients who are HP-negative, as in our case [9]. The present patient underwent upper gastrointestinal endoscopy during his first visit to the local hospital and was HP-negative on rapid urease testing performed at that time. Even after CP was suspected, the fecal HP antigen and serum HP antibody titers were negative. Our patient did not have HP infection, which is probably why HP eradication treatment was ineffective. As our patient was resistant to medical treatment and had diarrhea, bloody stools, and abdominal pain, he was finally treated surgically.

The general indication for surgery for CP is resistance to medical treatment, and symptoms such as diarrhea and abdominal pain improve quickly after surgery. However, previous reports have shown that recurrence is not uncommon in patients who have undergone surgery, and the surgical procedure performed varies depending on the extent of the lesions [2, 2]. Gallegos et al. performed right hemicolectomy to treat recurrence in the right colon after left hemicolectomy [3]. In the present case, CP was found from the anal canal to the middle of the transverse colon, and anastomosis between the transverse colon and the anus was difficult because of the large distance. Therefore, we performed IPAA.

Our patient’s symptoms improved rapidly after resection. No recurrence of CP was observed in the ileal pouch, the stoma was closed about 3 months later, and no recurrence has been observed in 1 year of follow-up. After total proctocolectomy, the number of stools may increase and soiling may occur due to changes in the stool properties, which may reduce quality of life. However, the anal function is often maintained after IPAA for ulcerative colitis [10, 11], and it is likely that the outcome will be the same after IPAA for different diseases. In the present case, the number of stools after surgery was about four per day and there was no soiling; the patient was satisfied with the outcome. There are reports that soiling after IPAA is more likely in patients older than 55 years [12, 12]. As our patient was in his 40 s, the preoperative anal sphincter function was maintained, which may have led to the good outcome after IPAA.

## Conclusion

We consider that laparoscopic-assisted total proctocolectomy and J-type IPAA with ileostomy may be a good option for CP, with little risk of recurrence.

## Data Availability

Not applicable.

## Abbreviations

CP: Cap polyposis
HP: *Helicobacter pylori*
TCS: Total colonoscopy
MNZ: Metronidazole
5-ASA: 5-Aminosalicylic acid
IPAA: Ileal pouch anal anastomosis
